# A novel oxidative stress-related genes signature associated with clinical prognosis and immunotherapy responses in clear cell renal cell carcinoma

**DOI:** 10.3389/fonc.2023.1184841

**Published:** 2023-08-03

**Authors:** Xin Wu, Fenghua Li, Wenjie Xie, Binbin Gong, Bin Fu, Weimin Chen, Libo Zhou, Lianmin Luo

**Affiliations:** ^1^ Department of Urology, The First Affiliated Hospital of Nanchang University, Nanchang, Jiangxi, China; ^2^ Department of Obstetrics and Gynecology, The First Affiliated Hospital of Nanchang University, Nanchang, Jiangxi, China

**Keywords:** clear cell renal cell carcinoma, oxidative stress, mRNA, prognosis signature, immune microenvironment, immunotherapy

## Abstract

**Background:**

Oxidative stress plays a significant role in the tumorigenesis and progression of tumors. We aimed to develop a prognostic signature using oxidative stress-related genes (ORGs) to predict clinical outcome and provide light on the immunotherapy responses of clear cell renal cell carcinoma (ccRCC).

**Methods:**

The information of ccRCC patients were collected from the TCGA and the E-MTAB-1980 datasets. Univariate Cox regression analysis and least absolute shrinkage and selection operator (LASSO) were conducted to screen out overall survival (OS)-related genes. Then, an ORGs risk signature was built by multivariate Cox regression analyses. The performance of the risk signature was evaluated with Kaplan-Meier (K-M) survival. The ssGSEA and CIBERSORT algorithms were performed to evaluate immune infiltration status. Finally, immunotherapy responses was analyzed based on expression of several immune checkpoints.

**Results:**

A prognostic 9-gene signature with *ABCB1*, *AGER*, *E2F1*, *FOXM1*, *HADH*, *ISG15*, *KCNMA1*, *PLG*, and *TEK*. The patients in the high risk group had apparently poor survival (TCGA: *p* < 0.001; E-MTAB-1980: *p* < 0.001). The AUC of the signature was 0.81 at 1 year, 0.76 at 3 years, and 0.78 at 5 years in the TCGA, respectively, and was 0.8 at 1 year, 0.82 at 3 years, and 0.83 at 5 years in the E-MTAB-1980, respectively. Independent prognostic analysis proved the stable clinical prognostic value of the signature (TCGA cohort: HR = 1.188, 95% CI =1.142-1.236, *p* < 0.001; E-MTAB-1980 cohort: HR =1.877, 95% CI= 1.377-2.588, *p* < 0.001). Clinical features correlation analysis proved that patients in the high risk group were more likely to have a larger range of clinical tumor progression. The ssGSEA and CIBERSORT analysis indicated that immune infiltration status were significantly different between two risk groups. Finally, we found that patients in the high risk group tended to respond more actively to immunotherapy.

**Conclusion:**

We developed a robust prognostic signature based on ORGs, which may contribute to predict survival and guide personalize immunotherapy of individuals with ccRCC.

## Introduction

Renal cell carcinoma (RCC) is a common malignant tumor of genitourinary system with an incidence only secondary to prostate cancer and bladder cancer, affecting nearly 431,000 new patients and 179,000 related deaths in 2020 worldwide (1). Clear cell renal cell carcinoma (ccRCC) is the most prevalent histological types of RCC, which accounting for about 70-80% of RCC (2). Clinically, approximately 30% of patients would experience tumor metastasis after curative surgical resection during the follow-up (3). The average amount of time from curative surgical resection to metastasis has not been reported in detail in the literature. There are no metastasis markers for ccRCC currently known. Imaging is used primarily to determine whether metastasis has occurred. In recent years, although targeted therapy and immunotherapy have greatly improved clinical outcome of patients with advanced ccRCC and become mainstays of treatment for advanced ccRCC (4, 5), however, a significant number of patients did not respond to these treatments (6). Due to heterogeneous disease, ccRCC patients with similar clinical condition may have distinctive prognosis. In clinical practice, it is a great challenge to early identify stratification of risk in patients with ccRCC and provide accurate clinical individualized therapeutic. With the development of sequencing, mounting evidences indicated that prognostic signature based on combining genes expression and clinical feature could be used as a new biomarker to optimize risk stratification, predict clinical prognosis and evaluate response to clinical treatment (7–9). These novel prognostic signatures could help doctors implemented individualized therapy to extend the survival of patients. Thus, establishing a reliable prognostic model is crucial to predict clinical prognosis and guide personal precision therapy for patients with ccRCC.

Oxidative stress is a common pathological phenomenon in the body, which is characterized by the imbalance between synthesis of oxidants and antioxidants, leading to the accumulation of large amounts of reactive oxygen species (ROS) (10). It has been reported that high levels of ROS contribute to tumorigenesis and tumor progression through a variety of pathways, such as tumor signaling pathways, tumor microenvironments, immune escape, metastasis, DNA mutations, and angiogenesis (11–14). Moreover, high levels of ROS also could affect the tumor development through influencing chemotherapeutic resistance and inducing cell apoptosis (15–17). Recent studies have shown that ORGs signature could be used as a biomarker for predicting clinical outcome and treatment responses in many cancers (18–21). In urologic cancer, ORGs signature was developed for predicting clinical outcome and immune status in patients with bladder cancer (22). Nevertheless, the clinical value of prognostic signature based on ORGs for ccRCC are needed investigated in depth.

In this study, a prognostic signature was established based on 9 ORGs to predict clinical outcome in individuals with ccRCC, and the effectiveness and reliability of the prognostic signature were further confirmed. In addition, immune cell infiltration and immunotherapy responses were comprehensively investigated.

## Materials and methods

**Figure 1:**
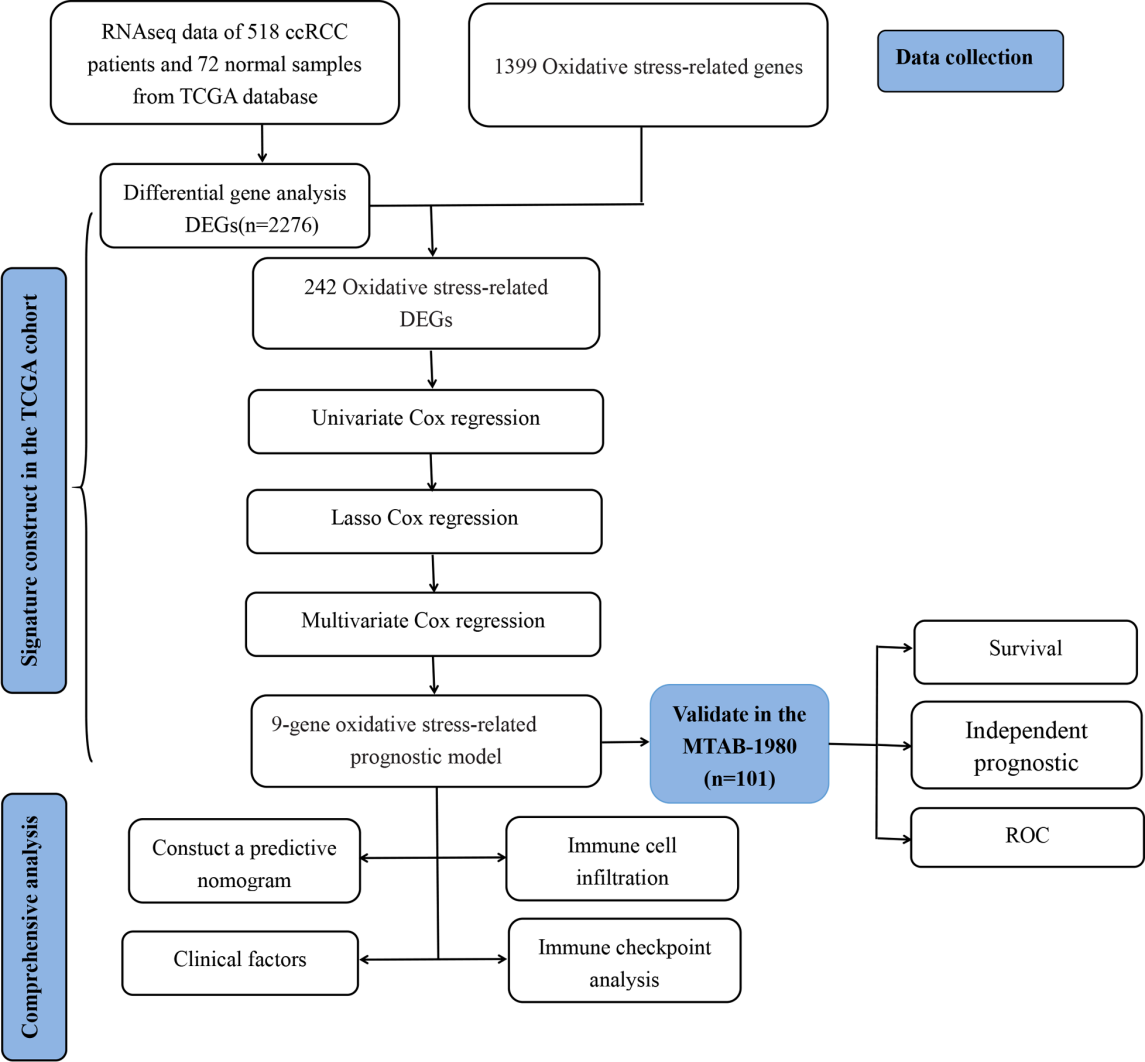
The flowchart of the present study design.

The complete procedures of this study was presented in **Figure 1**.

### Raw data collection

Transcripts and the corresponding clinical materials of ccRCC were acquired from the TCGA (https://genomecancer.ucsc.edu) and ArrayExpress datasets (E-MTAB-1980 dataset, https://www.ebi.ac.uk/arrayexpress/). Patients with survival less than one month or missing clinical information were excluded for our study.

### Identification of ORGs

ORGs were systematically searched from the GeneCards database (https://www.genecards.org/), with the screening threshold as relevance score ≥ 7. Finally, 1399 ORGs were included in our study (**Supplementary Table 1**).

### Screening for differentially expressed ORGs

Differentially expressed genes (DEGs) between tumor and normal tissues were analyzed by the Package “limma”, with the screening threshold as |log2FC| > 1 and adjusted *p* < 0.05.

### Establishment and validation of ORGs prognostic signature

Firstly, univariate Cox analysis was conducted to screen prognosis-related ORGs in the TCGA database. Next, the least absolute shrinkage and selection operator (LASSO) regression and multivariate Cox regression analysis were performed to screen genes for developing the prognostic signature for ccRCC. Subsequently, risk score was calculated with following formula:


$$\begin{array}{l} \text{risk score}={\text{β}}_{\text{mRNA}1}\times {\text{Expression}}_{\text{mRNA}1}+{\text{β}}_{\text{mRNA}2}\times {\text{Expression}}_{\text{mRNA}2}+{\text{β}}_{\text{mRNA}3}\\ \times {\text{Expression}}_{\text{mRNA}3}+\ldots +{\text{β}}_{\text{mRNAn}}\times {\text{Expression}}_{\text{mRNAn}}. \end{array}$$

According to the medium of risk score, individuals were classified into two groups (high risk group Vs low risk group). OS of the two groups was compared by K-M analysis. Finally, Receiver operating characteristic (ROC) curve was generated to assess the predictive accuracy and sensitivity of the prognostic signature.

### Establishment of a predictive nomogram

A nomogram was established containing risk score and independent prognostic factors based on results of the univariate and multivariate Cox regression analyses. The calibration curve was drawn to evaluate the predictive capability of nomogram by “rms” R package.

### Stratified analysis and comprehensive analysis of the ORGs signature

Stratified analysis was carried out to assess clinical value of the ORGs prognostic signature based on clinical features. In addition, to better evaluate the role of the ORGs signature in the ccRCC development, the differences in risk score were compared in different subgroups based on clinical features.

### Evaluation of tumor immune microenvironment

The ssGSEA algorithm was performed to calculate the infiltrating levels of 29 immune-related functional indicators, including immune cells and immune-related pathways. Next, the infiltration proportion of immune cell types was quantified with CIBERSORT algorithm.

### Evaluation of the response to immunotherapy

The expression level of four major immune checkpoints, including *PD-1*, *PD-L1*, *CTLA4* and *LAG3*, were analyzed in ccRCC tissues. Moreover, the association between risk score and the expression level of immune checkpoints was examined by the Spearman method.

### Statistical analysis

Statistical analyses were implemented by R software (version R-4.1.2) and GraphPad Prism (version 8.0.2). The difference in the continuous data between two groups was analyzed using Student’s t test. The correlation analysis was implemented by the Spearman method. A *p* value < 0.05 indicating a statistically significant (*p < 0.05, **p < 0.01, and ***p < 0.001).

## Results

### Identification of oxidative stress-related prognostic genes

**Figure 2:**
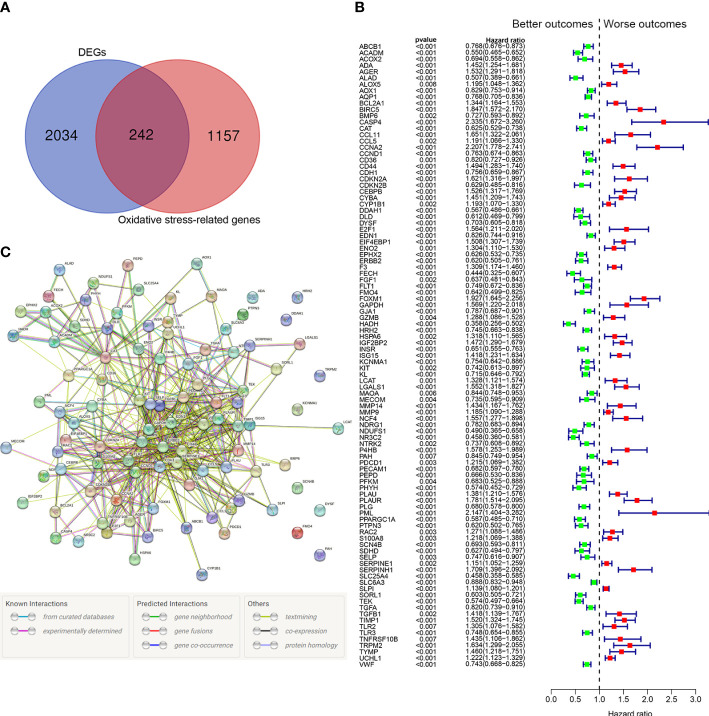
Identification of the oxidative stress-related prognostic genes in the TCGA cohort. **(A)** Venn diagram to identify differentially expressed ORGs between normal and tumor tissues. **(B)** Forest plots showing the significantly prognostic genes identified with univariate Cox regression analysis based on OS. The green colors represent better outcomes and red colors represent worse outcomes. **(C)** Interaction among candidate genes by PPI network.

Among 1399 ORGs, 242 DEGs were existed between tumor tissues and normal tissues (**Figure 2A**). Then, 99 ORGs associated with prognosis were identified based on the univariate Cox regression analysis (**Figure 2B**). Interactions of these 99 genes were further visualized with protein-protein interaction (PPI) network (**Figure 2C**).

### Establishment of the ORGs prognostic signature

**Figure 3:**
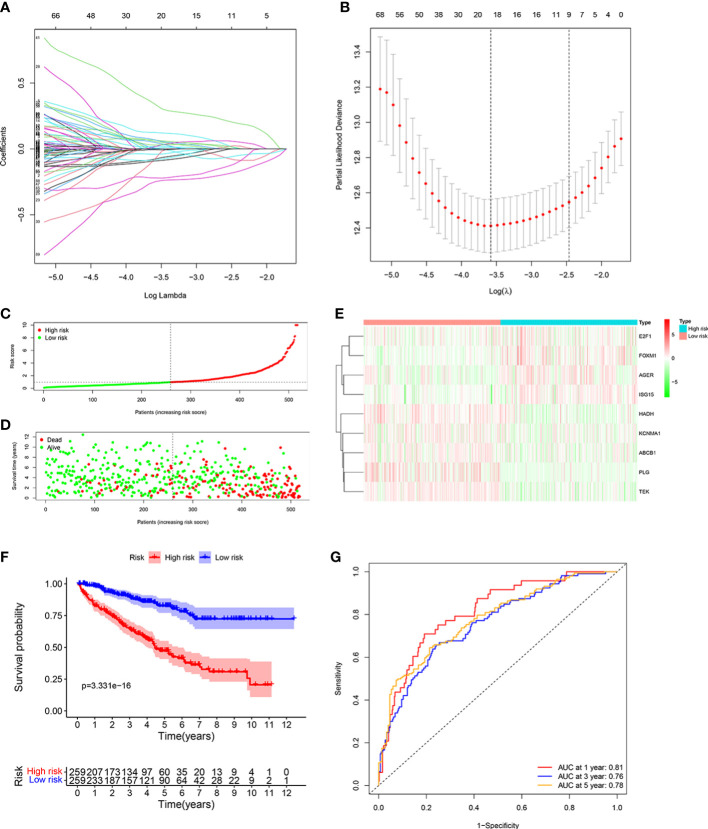
Developing the ORGs signature in the TCGA cohort. **(A**, **B)** LASSO regression analysis to select the optimal lambda and show optimal coefficients of the prognostic ORGs. **(C)** Distribution of the risk score in the low risk group and high risk group. **(D)** Distribution of survival status in the low risk group and high risk group. **(E)** Heatmap shows the expression of 9 signature genes in the low risk group and high risk group. **(F)** The K-M analysis shows the survival rate of patients in the low risk group and high risk group. **(G)** The ROC curve and AUC value of the ORGs signature for 1,3, and 5 years.

We identified 20 prognostic ORGs by Lasso Cox regression analysis (**Figures 3A, B**). Subsequently, multivariate Cox regression was performed to further filter out the candidate genes that were significantly related to survival, and finally identified 9 genes (*ABCB1*, *AGER*, *E2F1*, *FOXM1*, *HADH*, *ISG15*, *KCNMA1*, *PLG*, and *TEK*) for construction of prognostic model. The following equation was adopted to calculate risk score: risk score = (-0.148 × expression of *ABCB1*) + (0.169 × expression of *AGER*) + (-0.690 × expression of *E2F1*) + (0.704 × expression of *FOXM1*) + (-0.367 × expression of *HADH*) + (0.214 × expression of *ISG15*) + (-0.145 × expression of *KCNMA1*) + (-0.178 × expression of *PLG*) + (-0.394 × expression of *TEK*).

Patients were assigned into high risk group and low risk group based on the consideration of the median risk score (**Figure 3C**). The survival scatter chart revealed that individuals in the high risk group displayed a higher risk of mortality (**Figure 3D**). The expression level of *E2F1*, *FOXM1*, *AGER*, and *ISG15* were higher in the high risk group, while the expression level of *HADH*, *KCNMA1*, *ABCB1*, *PLG* and *TEK* were higher in the low risk group (**Figure 3E**). Compared to normal tissues, the expression level of *E2F1, FOXM1*, *AGER, ISG15*, and *KCNMA1* were significantly higher in ccRCC tissues, while the expression level of *HADH*, *ABCB1*, *PLG* and *TEK* were significantly lower (**Supplementary Figure 1**). We performed survival analysis on the 9 genes and found that patients with high expression level of identified genes, such as *E2F1*, *FOXM1*, *AGER* and *ISG15*, displayed a significantly poor outcome, while patients with high expression level of identified genes, such as *KCNMA1*, *HADH*, *ABCB1*, *PLG* and *TEK*, displayed a significantly favorable outcome (**Supplementary Figure 2**). K-M analysis shown that patients with low risk score displayed a significantly favorable outcome compared with patients with high risk score (**Figure 3F**). The area under the ROC curve (AUC) of 1 year (AUC=0.81), 3 years (AUC=0.76), and 5 years (AUC=0.78) were all larger than 0.70 (**Figure 3G**), suggesting that the prognostic signature displayed a favorable accuracy in predicting survival of patients with ccRCC.

### Validation of the ORGs signature in the E-MTAB-1980 cohort

**Figure 4:**
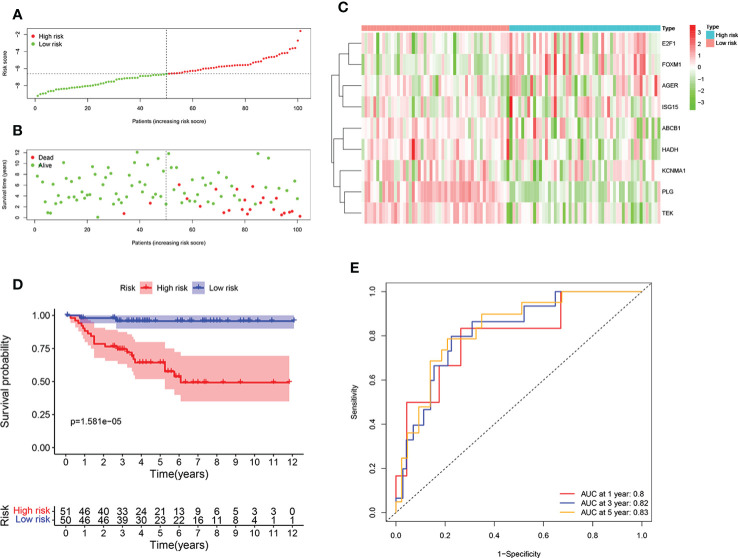
Validation of the ORGs signature in the E-MTAB-1980 dataset. **(A)** Distribution of the risk score in the low risk group and high risk group. **(B)** Distribution of survival status in the low risk group and high risk group. **(C)** Heatmap shows the expression of 9 signature genes in the low risk group and high risk group. **(D)** The K-M analysis shows the survival rate of patients in the low risk group and high risk group. **(E)** The ROC curve and AUC value of the ORGs signature for 1,3, and 5 years.

To further test the reliability of the ORGs prognostic signature, the same analyses were implemented in E-MTAB-1980 cohort. Individuals with high risk score had an adverse survival status (**Figures 4A, B**). Expression level pattern of these genes that make up the ORGs prognostic signature were consistent with those in the TCGA (**Figure 4C**). In addition, significantly shorter OS of patients with high risk score was observed (**Figure 4D**). The AUC for 1 year, 3 years, 5 years was 0.8, 0.82 and 0.83, respectively (**Figure 4E**), demonstrating that the ORGs prognostic signature displayed a good accuracy in predicting OS for patients with ccRCC.

### Stratified analysis

**Figure 5:**
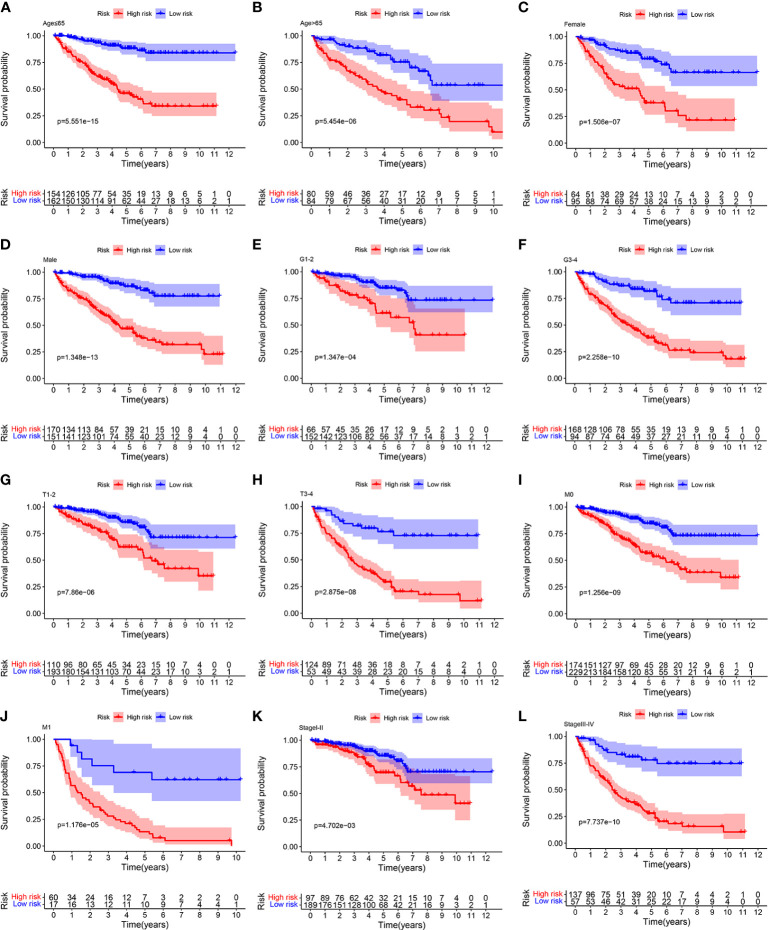
Relationship analysis of the ORGs signature and clinicopathological parameters. The survival rate of patients in the low risk group and high risk group among **(A, B)** age [ ≤ 65 years vs. >65 years], **(C, D)** gender [female vs. male], **(E, F)** tumor grade [G1–G2 vs. G3–G4], **(G, H)** T stage [T1–T2 vs. T3–T4], **(I, J)** M stage [M0 vs. M1], and **(K**, **L)** pathological stages [stageI–stageII vs. stageIII–stageIV].

To further confirm the accurately and independently prognostic value of the ORGs signature in ccRCC, stratification analysis was performed in different subgroups based on clinical features. We found that significantly poor OS was observed in the high risk group in all subgroups, such as age (**Figures 5A, B**), gender (**Figures 5C, D**), tumor grade (**Figures 5E, F**), T stage (**Figures 5G, H**), M stage (**Figures 5I, J**), and pathological stage (**Figures 5K, L**). These findings suggested that the ORGs signature had universal applicability in predicting OS for patients with ccRCC.

### Correlation of the ORGs signature with clinical features

**Figure 6:**
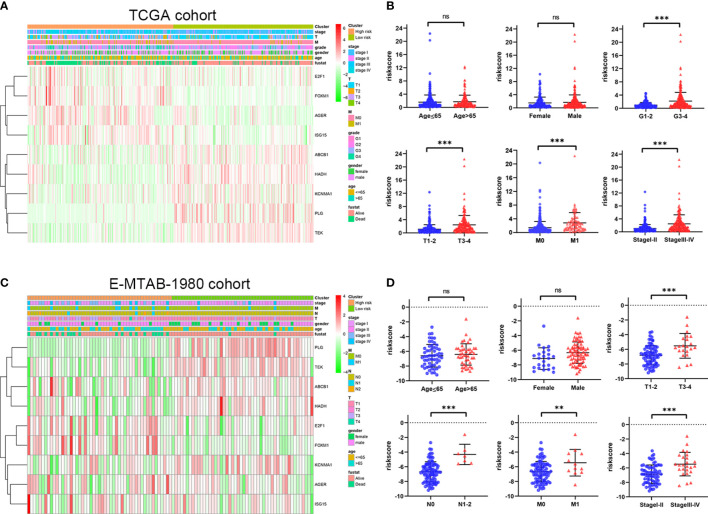
Relationship between risk score of the ORGs signature and clinicopathological parameters. **(A)** The heatmap shows the distribution of clinicopathological parameters and 9 signature genes in the TCGA cohort. **(B)** The distribution of risk score among different clinicopathological parameters in the TCGA cohort. **(C)** The heatmap shows the distribution of clinicopathological parameters and 9 signature genes in the E-MTAB-1980 cohort. **(D)** The distribution of risk score among different clinicopathological parameters in the E-MTAB-1980 cohort. *p* values were shown as: ns, not significant; **, *p*< 0.01; ***, *p*< 0.001.

To further explore the correlation between the ORGs signature and clinical features, risk score was calculated in the different subgroups based on clinical features. From the TCGA cohort analysis results, it was shown that risk score was significantly higher in G3-4, T3-4, M1, and stageIII-IV subgroups than those in the corresponding early clinicopathological stage subgroups (**Figures 6A, B**). In addition, risk score of patients in the E-MTAB-1980 cohort was significantly higher in T3-4, N1-2, M1, and stageIII-IV subgroups than those in the corresponding early clinicopathological stage subgroups (**Figures 6C, D**). These results indicated that ORGs signature might act as a malignancy biomarker for ccRCC.

### Independence of the ORGs signature and nomogram construction

**Figure 7:**
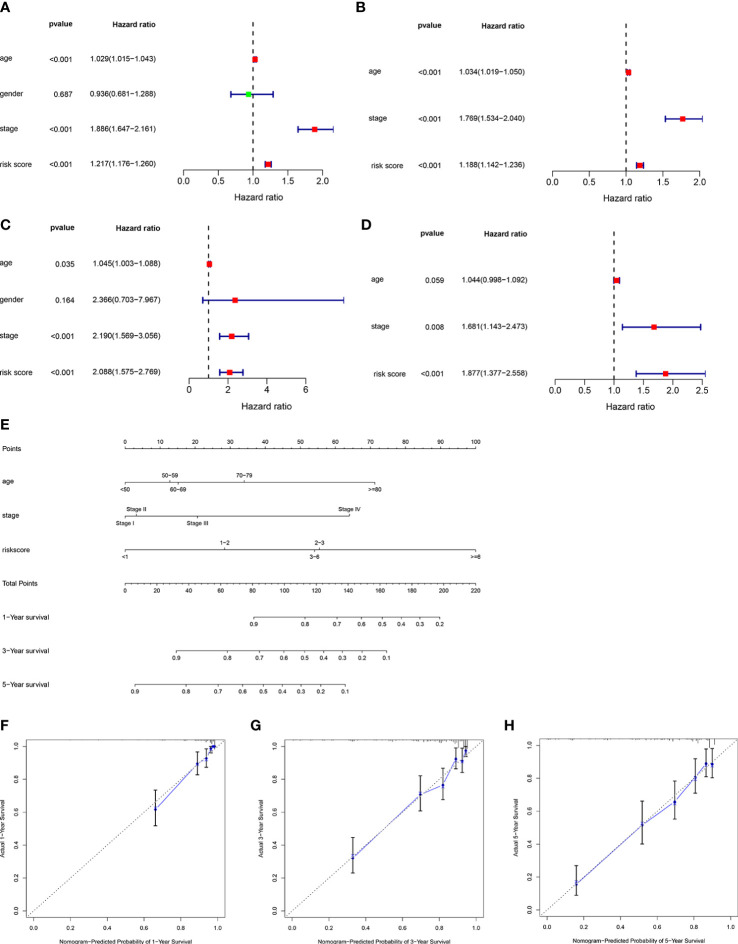
Construction of a nomogram predicting OS in ccRCC. Univariate **(A)** and multivariate **(B)** Cox regression analysis of the clinicopathological features in the TCGA cohort. Univariate **(C)** and multivariate **(D)** Cox regression analysis of the clinicopathological features in the E-MTAB-1980 cohort. **(E)** Construction of the nomogram using risk score, age and stage. **(F–H)** Calibration curve of nomogram for predicting survival at 1, 3 and 5 years.

To further confirm whether risk score could be used as an independent prediction biomarker for ccRCC survival, univariate and multivariate Cox regression analyses were implemented. Results demonstrated that risk score of the ORGs signature could be used as an independent indicator for predicting survival of ccRCC (**Figures 7A–D**). Additionally, nomogram was widely used in predicting OS of patients with cancer based on nomogram scores, therefore, a nomogram was constructed by integrating independent prognostic markers based on multivariate Cox regression analysis (**Figure 7E**). The calibration curve illustrated that the nomogram had an excellent capability in predicting survival at 1, 3, and 5 years (**Figures 7F–H**).

### Relationship between the ORGs signature and immune infiltration in the TCGA cohort

**Figure 8:**
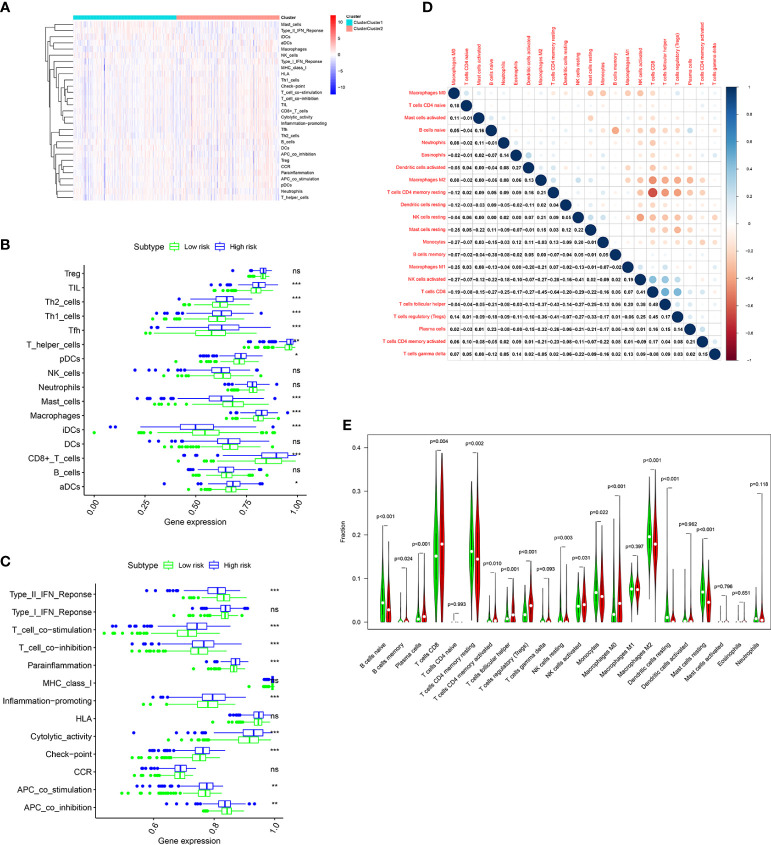
Immune cell infiltration analysis in the TCGA cohort. **(A)** Heatmap shows the distribution of 16 immune cells and activity of 13 immune-related pathways in the low risk group and high risk group based on ssGSEA algorithm. The potential differences of 16 immune cells **(B)** and 13 immune-related pathways **(C)** between the low risk group and high risk group based on ssGSEA algorithm. **(D)** Correlation among 22 immune cell types using CIBERSORT algorithm. **(E)** Violin plot shows potential differences of 22 immune cell infiltration between the low risk group and high risk group using CIBERSORT algorithm. *p* values were shown as: ns, not significant; *, *p*< 0.05; **, *p*< 0.01; ***, *p*< 0.001.

The immune infiltration landscape was explored in patients by multiple algorithms. The sGSEA algorithm results shown that infiltration abundance of most immune cells, including TIL, Th2_cells, Th1_cell, Tfh, T_helper_cells, pDCs, Macrophages, CD8+_T_cells, and aDCs, were considerably higher in individuals in the high risk group, whereas infiltration abundance of Mast_cells and iDCs were obviously higher in individuals in the low risk group (**Figures 8A, B**). Additionally, significantly higher immune function scores, such as T_cell_costimulation, T_cell_co-inhibition, Parainflammation, Inflammation-promoting, Cytolytic_activity, check-point, and APC_co_stimulation, were observed in individuals in the high risk group (**Figure 8C**). Meanwhile, the CIBERSORT algorithm was performed to analyze infiltration abundance of 22 types of immune cells in patients. Correlations of these immune cells were shown in **Figure 8D**. As shown in **Figure 8E**, significantly higher abundance of B cells memory, Plasma cells, T cells CD8, T cells CD4 memory activated, T cells follicular helper, T cells regulatory, NK cells activated, and Macrophages M0 were observed in individuals in the high risk group, however, significantly higher abundance of B cells naive, T cells CD4 memory resting, NK cells resting, Monocytes, Macrophages M2, Dendritic cells resting, and Mast cells resting were observed in individuals in the low risk group.

### Relationship between the ORGs signature and immunotherapy response

**Figure 9:**
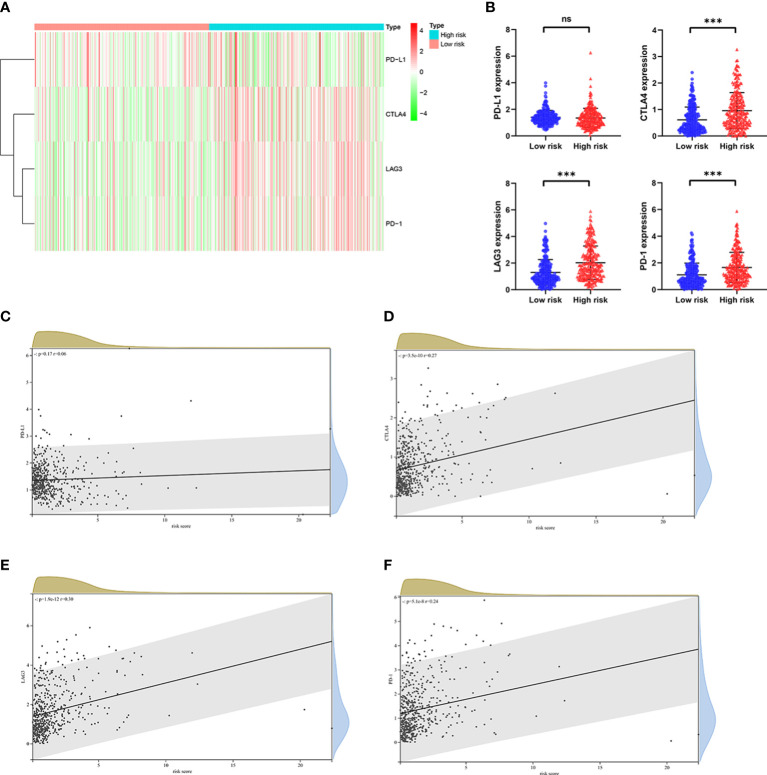
The relationship between risk score and expression of immune checkpoint genes in the TCGA cohort. **(A)** Heatmap shows the expression of immune checkpoint genes, including *PD-1*, *CTLA4*, *LAG3*, and *PD-L1* in the low risk group and high risk group. **(B)** The potential differences in *PD-1*, *CTLA4*, *LAG3*, and *PD-L1* expression between the low risk group and high risk group. The correlation between **(C)** *PD-1*, **(D)** *CTLA4*, **(E)** *LAG3*, and **(F)** *PD-L1* expression and risk score in ccRCC. p values were shown as: ns, not significant; ***, *p*< 0.001.

Numerous studies have shown that immunotherapy is one of the effective treatments for patients with advanced ccRCC, and high expression level of immune checkpoint genes related to a better response to immunotherapy (3–5). The expression level of some well-known immune checkpoint genes, including *PD-1*, *PD-L1*, *CTLA4* and *LAG3* were analyzed in patients from TCGA cohort. As shown in **Figures 9A, B**, considerably higher expression level of *CTLA4*, *LAG3* and *PD-*1 were observed in patients in the high risk group, whereas the expression level of *PD-L1* was no substantial change between the two groups. As predicted, the expression level of *CTLA4*, *LAG3* and *PD-1* were positively correlated with the risk score (**Figures 9C–F**). Taken together, these findings illustrated that patients with high risk score had a higher sensitivity for immunotherapy responses.

## Discussion

Oxidative stress, caused by the accumulation of large amounts of ROS, plays a vital role in the several phases of development of tumors, such as tumor initiation, progression and metastasis (10). In addition, accumulating evidences indicated that oxidative stress not only associated with tumor progression but also affected both the tumor microenvironment and the immunotherapy responses (23–25). In recent years, many studies focused on constructing prognostic signature for predicting prognosis and sensitivity to treatment of cancers (18–21). Hence, constructing a risk prognostic signature using ORGs for predicting clinical outcome of ccRCC may be promising.

We built a prognostic signature using 9 ORGs, and finally confirmed its clinical value. We identified two risk groups based on the calculated risk score in patients with ccRCC, and observed that survival of patients in the high risk group was obviously shorter than that of patients in the low risk group. The predicting efficiency of the signature was verified using ROC and independent prognostic analysis. The results suggested that the ORGs signature had accurately and independently predictive ability. Further, stratification analysis was performed in different subgroups based on clinical features, which suggested that patients in the high risk group had poor outcome in all subgroups. Meanwhile, we also found that patients in the high risk group were correlated with worse clinical features in terms of the tumor grade, T stage, M stage, N stage, and pathological stage. In addition, we built a nomogram model according to the results of multivariate independent prognostic analysis; high predictive performance was observed from the calibration graph.

Our signature consisted of 9 ORGs, including *ABCB1*, *AGER*, *E2F1*, *FOXM1*, *HADH*, *ISG15*, *KCNMA1*, *PLG*, and *TEK*. *ABCB1* was designed to protect cells from damage caused by xenobiotic and toxic substances, including chemotherapy drugs (26). It was reported that *ABCB1* could confer resistance to chemotherapy (27). A recent review reported that inhibiting *ABCB1* could restore cancer cell susceptibility to chemotherapy drugs (28). *AGER* was a member of immunoglobulin superfamily of cell surface receptors. *AGER* expression was associated with immune inflammatory response and cancerogenesis (29). Many evidences shown that the expression level and mutation rate of *AGER* were increased in multiple cancers, such as esophageal cancer, breast cancer, gastric cancer and endometrial cancer (30–33). *E2F1*, a member of *E2F* transcription factor family, was up-regulated and confirmed as an oncogene in multiple human cancers, including hepatocellular cancer, breast cancer and gastric cancer (34–36). Shen et al. found that expression of *E2F1* was up-regulated in ccRCC, and *E2F1* knockdown inhibited the proliferation and metastasis of ccRCC cells (37). The transcriptional factor Forkhead Box M1 (*FOXM1*) was reported to belongs to the Forkhead box (FOX) transcription factor family (38). Previous studies revealed that *FOXM1* transcription could increase the expression of multiple genes important for cancers progression (39–41). Jiang et al. found that *FOXM1* regulated *LINC01094* expression to promote ccRCC progression (42). *HADH* was a very important enzyme in the β-oxidation of fatty acid (43). It was reported that *HADH* expression was down-regulated in many tumors and its low expression was associated with tumors progression (44, 45). *ISG15* (IFN-stimulated gene), a ubiquitin-like protein, has been shown to be a tumor-related gene involved in tumors pathogenesis (46, 47). In cervical cancer, *ISG15* expression was up-regulated and knockdown *ISG15* inhibited proliferation and invasion of cervical cancer cell (48). *KCNMA1* was one of the high voltage-activated channel conductance for potassium ions (49). Previous studies reported that *KCNMA1* may be involved in various human tumorigenesis processes, such as prostate cancer (50), breast cancer (51), cervical cancer (52), and colorectal cancer (53). Plasminogen (*PLG*) acted as an important role in inhibiting tumor progression due to its ability to inhibit angiogenesis (54). *PLG* expression was down-regulated in ccRCC, and its low expression was associated with poor clinical outcome (55). *TEK*, also known as *TIE-2*, was a tyrosine kinase receptor for endothelial cells, with the ability to regulate of angiogenesis and remodeling (56). Liao et al. (57) found that TEK was low expressed in ccRCC compared with normal tissues and downregulation of TEK correlated with a poor clinical outcome which was also confirmed in previous studies (58, 59).

Immune infiltration was closely related to tumor progression and immunotherapy responses. Thus, we further evaluated the relationship between the signature and immune infiltration status of ccRCC. Results from ssGSEA algorithm indicated that compared with individuals in the low risk group, individuals in the high risk group displayed more accumulation of immune cell infiltration and higher activity of immunity-related pathways. The results of CIBERSORT algorithm shown that infiltration abundance of B cells memory, Plasma cells, T cells CD8, T cells CD4 memory activated, T cells follicular helper, T cells regulatory, NK cells activated, and Macrophages M0 were obviously higher in individuals with high risk score, while infiltration abundance of B cells naive, T cells CD4 memory resting, NK cells resting, Monocytes, Macrophages M2, Dendritic cells resting, and Mast cells resting were more higher in individuals with low risk score. Both T cells regulatory and T cells CD8 infiltration were related to adverse clinical outcome in individuals with ccRCC (60–62). Zhang et al. found that ccRCC patients with favorable prognosis presented relatively higher enrichment levels of B cells naive, T cells CD4 memory resting, NK cells resting, Monocytes and macrophages M2 (63).

Immunotherapy had proven to be effective and could significantly improve the prognosis for patients with advanced ccRCC (3–5). In our study, patients in the high risk group presented significantly higher expression level of *CTLA4*, *LAG3* and *PD-1*. Of note, the above mentioned immune checkpoint genes expression were all positively associated with risk score of the signature. These results implied that compared with patients with low risk score, patients with high risk score displayed higher sensitivity for immunotherapy, and thus, the ORGs signature might have a potential in guiding personalized immunotherapy for patients with advanced ccRCC.

A recent article by Ma et al. also reported that an oxidative stress signature predicted clinical outcome in ccRCC and 4 oxidative stress genes (*UCN*, *PLG*, *FOXM1*, *HRH2*) were selected to construct a prognostic signature (64). The AUC of the 4 oxidative stress genes prognostic signature was 0.77 at 1 year, 0.70 at 3 years, and 0.71 at 5 years in the TCGA. Zhang et al. constructed a signature based on oxidative-stress related lncRNA in ccRCC (65). The signature consisted of 7 lncRNAs, including *SPART-AS1*, *AL162586.1*, *LINC00944*, *LINC01550*, *HOXB-AS4*, *LINC02027*, and *DOCK9-DT*. Wu et al. constructed a signature using mitochondrial genes related to oxidative stress to predict clinical outcome for ccRCC in the TCGA (66). Further analysis identified 6 prognostic-related mitochondrial genes, including *ACAD11*, *ACADSB*, *BID*, *PYCR1*, *SLC25A27*, *and STAR.* The AUC of the 6 mitochondrial genes prognostic signature was 0.736 at 1 year, 0.707 at 3 years, and 0.758 at 5 years in the TCGA. In our study, we constructed a prognostic signature using 9 four oxidative stress genes (*ABCB1*, *AGER*, *E2F1*, *FOXM1*, *HADH*, *ISG15*, *KCNMA1*, *PLG*, *TEK*). The AUC of the 9 oxidative stress genes prognostic signature was 0.81 at 1 year, 0.76 at 3 years, and 0.78 at 5 years in the TCGA.

Nonetheless, some limitations were presented in our study. First, our conclusions were obtained based on bioinformatic analysis, and multicenter and large-cohort clinical trials validation are needed to verify the robustness of the prognostic signature. Second, more in-depth experiments about the detailed biological functions of these genes that make up the ORGs signature are necessary to be investigated.

## Conclusions

A novel 9-gene signature was built on the basis of the ORGs for predicting the clinical prognosis of ccRCC. It was proven that the prognosis signature model had a good and independent prediction performance. In addition, the prognosis signature might have a potential in predicting the responses of immunotherapy for patients with ccRCC, which could help clinicians to make immunotherapy decisions in order to achieve personalized treatment.

## Data availability statement

The original contributions presented in the study are included in the article/**Supplementary Material**. Further inquiries can be directed to the corresponding author.

## Author contributions

LL and XW contributed to the study conceptualization and design. FL and WX contributed to statistical analysis. BG, BF, WC, and LZ performed data collection. LL and XW composed the manuscript. All authors reviewed the submitted version. All authors contributed to the article and approved the submitted version.

## Supplementary material

Supplementary Figure 1
**(A–I)** The expression levels of the signature genes (*ABCB1*, *AGER*, *E2F1*, *FOXM1*, *HADH*, *ISG15*, *KCNMA1*, *PLG*, and *TEK*) in ccRCC tissues compared with normal tissues in the TCGA data.

Supplementary Figure 2
**(A–I)** The survival plot for the signature genes (*ABCB1*, *AGER*, *E2F1*, *FOXM1*, *HADH*, *ISG15*, *KCNMA1*, *PLG*, and *TEK*) in the TCGA data.

## References

[1] Sung H Ferlay J Siegel RL Laversanne M Soerjomataram I Jemal A. Global cancer statistics 2020: GLOBOCAN estimates of incidence and mortality worldwide for 36 cancers in 185 countries. CA Cancer J Clin (2021) 71(3):209–49. doi: 10.3322/caac.21660 33538338

[2] Ljungberg B Albiges L Abu-Ghanem Y Bedke J Capitanio U Dabestani S. European association of urology guidelines on renal cell carcinoma: the 2022 update. Eur Urol. (2022) 82(4):399–410. doi: 10.1016/j.eururo.2022.03.006 35346519

[3] Motzer RJ Hutson TE Cella D Reeves J Hawkins R Guo J. Pazopanib versus sunitinib in metastatic renal-cell carcinoma. N Engl J Med (2013) 369(8):722–31. doi: 10.1056/NEJMoa1303989 23964934

[4] Motzer RJ Powles T Burotto M Escudier B Bourlon MT Shah AY. Nivolumab plus cabozantinib versus sunitinib in first-line treatment for advanced renal cell carcinoma (CheckMate 9ER): long-term follow-up results from an open-label, randomised, phase 3 trial. Lancet Oncol (2022) 23(7):888–98. doi: 10.1016/S1470-2045(22)00290-X PMC10305087 35688173

[5] Rini BI Plimack ER Stus V Gafanov R Hawkins R Nosov D. Pembrolizumab plus Axitinib versus Sunitinib for Advanced Renal-Cell Carcinoma. N Engl J Med (2019) 380(12):1116–27. doi: 10.1056/NEJMoa1816714 30779529

[6] Sharma R Kadife E Myers M Kannourakis G Prithviraj P Ahmed N. Determinants of resistance to VEGF-TKI and immune checkpoint inhibitors in metastatic renal cell carcinoma. J Exp Clin Cancer Res (2021) 40(1):186. doi: 10.1186/s13046-021-01961-3 34099013 PMC8183071

[7] Cheong JH Wang SC Park S Porembka MR Christie AL Kim H. Development and validation of a prognostic and predictive 32-gene signature for gastric cancer. Nat Commun (2022) 13(1):774. doi: 10.1038/s41467-022-28437-y 35140202 PMC8828873

[8] Liu Z Qi T Li X Yao Y Othmane B Chen J. A novel TGF-β Risk score predicts the clinical outcomes and tumour microenvironment phenotypes in bladder cancer. Front Immunol (2021) 12:791924. doi: 10.3389/fimmu.2021.791924 34975891 PMC8718409

[9] Yang Y Yang Y Liu J Zeng Y Guo Q Guo J. Establishment and validation of a carbohydrate metabolism-related gene signature for prognostic model and immune response in acute myeloid leukemia. Front Immunol (2022) 13:1038570. doi: 10.3389/fimmu.2022.1038570 36544784 PMC9761472

[10] Hayes JD Dinkova-Kostova AT Tew KD. Oxidative stress in cancer. Cancer Cell (2020) 38(2):167–97. doi: 10.1016/j.ccell.2020.06.001 PMC7439808 32649885

[11] Aggarwal V Tuli HS Varol A Thakral F Yerer MB Sak K. Role of reactive oxygen species in cancer progression: molecular mechanisms and recent advancements. Biomolecules (2019) 9(11):735. doi: 10.3390/biom9110735 31766246 PMC6920770

[12] Cheung EC Vousden KH. The role of ROS in tumour development and progression. Nat Rev Cancer (2022) 22(5):280–97. doi: 10.1038/s41568-021-00435-0 35102280

[13] Renaudin X. Reactive oxygen species and DNA damage response in cancer. Int Rev Cell Mol Biol (2021) 364:139–61. doi: 10.1016/bs.ircmb.2021.04.001 34507782

[14] Sarmiento-Salinas FL Perez-Gonzalez A Acosta-Casique A Ix-Ballote A Diaz A Treviño S. Reactive oxygen species: Role in carcinogenesis, cancer cell signaling and tumor progression. Life Sci (2021) 284:119942. doi: 10.1016/j.lfs.2021.119942 34506835

[15] Bhardwaj V He J. Reactive oxygen species, metabolic plasticity, and drug resistance in cancer. Int J Mol Sci (2020) 21(10):3412. doi: 10.3390/ijms21103412 32408513 PMC7279373

[16] Cui Q Wang JQ Assaraf YG Ren L Gupta P Wei L. Modulating ROS to overcome multidrug resistance in cancer. Drug Resist Updat. (2018) 41:1–25. doi: 10.1016/j.drup.2018.11.001 30471641

[17] Yu G Luo H Zhang N Wang Y Li Y Huang H. Loss of p53 sensitizes cells to palmitic acid-induced apoptosis by reactive oxygen species accumulation. Int J Mol Sci (2019) 20(24):6268. doi: 10.3390/ijms20246268 31842349 PMC6941153

[18] Lu W Yin C Zhang T Wu Y Huang S. An oxidative stress-related prognostic signature for indicating the immune status of oral squamous cell carcinoma and guiding clinical treatment. Front Genet (2022) 13:977902. doi: 10.3389/fgene.2022.977902 36212161 PMC9538189

[19] Wang L Liu X. An oxidative stress-related signature for predicting the prognosis of liver cancer. Front Genet (2023) 13:975211. doi: 10.3389/fgene.2022.975211 36685933 PMC9845401

[20] Peng H Li X Luan Y Wang C Wang W. A novel prognostic model related to oxidative stress for treatment prediction in lung adenocarcinoma. Front Oncol (2023) 13:1078697. doi: 10.3389/fonc.2023.1078697 36798829 PMC9927401

[21] Liu Q Yang X Yin Y Zhang H Yin F Guo P. Identifying the role of oxidative stress-related genes as prognostic biomarkers and predicting the response of immunotherapy and chemotherapy in ovarian cancer. Oxid Med Cell Longev (2022) 2022:6575534. doi: 10.1155/2022/6575534 36561981 PMC9764017

[22] Zhang M Du G Li Z Li D Li W Li H. An oxidative stress-related genes signature for predicting survival in bladder cancer: based on TCGA database and bioinformatics. Int J Gen Med (2022) 15:2645–67. doi: 10.2147/IJGM.S348945 PMC8922338 35300137

[23] Aboelella NS Brandle C Kim T Ding ZC Zhou G. Oxidative stress in the tumor microenvironment and its relevance to cancer immunotherapy. Cancers (Basel). (2021) 13(5):986. doi: 10.3390/cancers13050986 33673398 PMC7956301

[24] Kirtonia A Sethi G Garg M. The multifaceted role of reactive oxygen species in tumorigenesis. Cell Mol Life Sci (2020) 77(22):4459–83. doi: 10.1007/s00018-020-03536-5 PMC11105050 32358622

[25] Kotsafti A Scarpa M Castagliuolo I Scarpa M. Reactive oxygen species and antitumor immunity-from surveillance to evasion. Cancers (Basel) (2020) 12(7):1748. doi: 10.3390/cancers12071748 32630174 PMC7409327

[26] Kathawala RJ Gupta P Ashby CR Jr Chen ZS. The modulation of ABC transporter-mediated multidrug resistance in cancer: a review of the past decade. Drug Resist Updat. (2015) 18:1–17. doi: 10.1016/j.drup.2014.11.002 25554624

[27] Robey RW Pluchino KM Hall MD Fojo AT Bates SE Gottesman MM. Revisiting the role of ABC transporters in multidrug-resistant cancer. Nat Rev Cancer. (2018) 18(7):452–64. doi: 10.1038/s41568-018-0005-8 PMC6622180 29643473

[28] Engle K Kumar G. Cancer multidrug-resistance reversal by ABCB1 inhibition: A recent update. Eur J Med Chem (2022) 239:114542. doi: 10.1016/j.ejmech.2022.114542 35751979

[29] Bongarzone S Savickas V Luzi F Gee AD. Targeting the receptor for advanced glycation endproducts (RAGE): A medicinal chemistry perspective. J Med Chem (2017) 60:7213–32. doi: 10.1021/acs.jmedchem.7b00058 PMC5601361 28482155

[30] Jing RR Cui M Sun BL Yu J Wang HM. Tissue-specific expression profiling of receptor for advanced glycation end products and its soluble forms in esophageal and lung cancer. Genet Test Mol Biomarkers. (2010) 14:355–61. doi: 10.1089/gtmb.2009.0064 20578941

[31] Nankali M Karimi J Goodarzi MT Saidijam M Khodadadi I Razavi AN. Increased expression of the receptor for advanced glycation end-products (RAGE) is associated with advanced breast cancer stage. Oncol Res Treat (2016) 39:622–8. doi: 10.1159/000449326 27710974

[32] Wang D Li T Ye G Shen Z Hu Y Mou T. Overexpression of the receptor for advanced glycation endproducts (RAGE) is associated with poor prognosis in gastric cancer. PloS One (2015) 10:e0122697. doi: 10.1371/journal.pone.0122697 25860956 PMC4393278

[33] Zheng L Li D Zhou YM Yang H Cheng D Ma XX. Effects of receptor for advanced glycation endproducts on microvessel formation in endometrial cancer. BMC Cancer. (2016) 16:93. doi: 10.1186/s12885-016-2126-3 26873694 PMC4751660

[34] Qiao L Zhang Q Sun Z Liu Q Wu Z Hu W. The E2F1/USP11 positive feedback loop promotes hepatocellular carcinoma metastasis and inhibits autophagy by activating ERK/mTOR pathway. Cancer Lett (2021) 514:63–78. doi: 10.1016/j.canlet.2021.05.015 34044068

[35] Ma J He Z Zhang H Zhang W Gao S Ni X. SEC61G promotes breast cancer development and metastasis via modulating glycolysis and is transcriptionally regulated by E2F1. Cell Death Dis (2021) 12(6):550. doi: 10.1038/s41419-021-03797-3 34039955 PMC8155024

[36] Chen YU Yu Y Lv M Shi Q Li X. E2F1-mediated up-regulation of TOP2A promotes viability, migration, and invasion, and inhibits apoptosis of gastric cancer cells. J Biosci (2022) 47:84. doi: 10.1007/s12038-022-00322-2 36550695

[37] Shen D Gao Y Huang Q Xuan Y Yao Y Gu L. E2F1 promotes proliferation and metastasis of clear cell renal cell carcinoma via activation of SREBP1-dependent fatty acid biosynthesis. Cancer Lett (2021) 514:48–62. doi: s 10.1016/j.canlet.2021.05.012 34019961

[38] Barger CJ Branick C Chee L Karpf AR. Pan-cancer analyses reveal genomic features of FOXM1 overexpression in cancer. Cancers (Basel). (2019) 11(2):251. doi: 10.3390/cancers11020251 30795624 PMC6406812

[39] Yi L Wang H Li W Ye K Xiong W Yu H. The FOXM1/RNF26/p57 axis regulates the cell cycle to promote the aggressiveness of bladder cancer. Cell Death Dis (2021) 12(10):944. doi: 10.1038/s41419-021-04260-z 34650035 PMC8516991

[40] Zhou DM Liu J Liu F Luo GW Li HT Zhang R. A novel FoxM1-PSMB4 axis contributes to proliferation and progression of cervical cancer. Biochem Biophys Res Commun (2020) 521(3):746–52. doi: 10.1016/j.bbrc.2019.10.183 31699366

[41] Kimura H Sada R Takada N Harada A Doki Y Eguchi H. The Dickkopf1 and FOXM1 positive feedback loop promotes tumor growth in pancreatic and esophageal cancers. Oncogene (2021) 40(26):4486–502. doi: 10.1038/s41388-021-01860-z PMC8249240 34117362

[42] Jiang Y Zhang H Li W Yan Y Yao X Gu W. FOXM1-activated LINC01094 promotes clear cell renal cell carcinoma development via microRNA 224-5p/CHSY1. Mol Cell Biol (2020) 40(3):e00357–19. doi: 10.1128/MCB.00357-19 PMC6965037 31767633

[43] Fang H Li H Zhang H Wang S Xu S Chang L. Short-chain L-3-hydroxyacyl-CoA dehydrogenase: A novel vital oncogene or tumor suppressor gene in cancers. Front Pharmacol (2022) 13:1019312. doi: 10.3389/fphar.2022.1019312 36313354 PMC9614034

[44] Shen C Song YH Xie Y Wang X Wang Y Wang C. Downregulation of HADH promotes gastric cancer progression via Akt signaling pathway. Oncotarget (2017) 8(44):76279–89. doi: 10.18632/oncotarget.19348 PMC5652705 29100311

[45] Jiang H Chen H Wan P Chen N. Decreased expression of HADH is related to poor prognosis and immune infiltration in kidney renal clear cell carcinoma. Genomics (2021) 113(6):3556–64. doi: 10.1016/j.ygeno.2021.08.008 34391866

[46] Wood LM Pan ZK Seavey MM Muthukumaran G Paterson Y. The ubiquitin-like protein, ISG15, is a novel tumor-associated antigen for cancer immunotherapy. Cancer Immunol Immunother. (2012) 61:689–700. doi: 10.1007/s00262-011-1129-9 22057675 PMC4561532

[47] Kiessling A Hogrefe C Erb S Bobach C Fuessel S Wessjohann L. Expression, regulation and function of the ISGylation system in prostate cancer. Oncogene (2009) 28:2606–20. doi: 10.1038/onc.2009.115 19430494

[48] Tao P Sun L Sun Y Wang Y Yang Y Yang B. ISG15 is associated with cervical cancer development. Oncol Lett (2022) 24(4):380. doi: 10.3892/ol.2022.13500 36238852 PMC9494601

[49] Ge L Hoa NT Wilson Z Arismendi-Morillo G Kong X-T Tajhya RB. Big Potassium (BK) ion channels in biology, disease and possible targets for cancer immunotherapy. Int Immunopharmacol. (2014) 22:427–43. doi: 10.1016/j.intimp.2014.06.040 PMC5472047 25027630

[50] Bloch M Ousingsawat J Simon R Schraml P Gasser TC Mihatsch MJ. KCNMA1 gene amplification promotes tumor cell proliferation in human prostate cancer. Oncogene (2007) 26:2525–34. doi: 10.1038/sj.onc.1210036 17146446

[51] Khaitan D Sankpal UT Weksler B Meister EA Romero IA Couraud PO. Role of KCNMA1 gene in breast cancer invasion and metastasis to brain. BMC Cancer. (2009) 9:258. doi: 10.1186/1471-2407-9-258 19640305 PMC2727533

[52] Ramírez A Vera E Gamboa-Domínguez A Lambert P Gariglio P Camacho J. Calcium-activated potassium channels as potential early markers of human cervical cancer. Oncol Lett (2018) 15:7249–54. doi: 10.3892/ol.2018.8187 PMC5920501 29725443

[53] Basile MS Fagone P Mangano K Mammana S Magro G Salvatorelli L. KCNMA1 expression is downregulated in colorectal cancer via epigenetic mechanisms. Cancers (Basel) (2019) 11(2):245. doi: 10.3390/cancers11020245 30791468 PMC6406553

[54] Ismail AA Shaker BT Bajou K. The plasminogen-activator plasmin system in physiological and pathophysiological angiogenesis. Int J Mol Sci (2021) 23(1):337. doi: 10.3390/ijms23010337 35008762 PMC8745544

[55] Zhang Z Lin E Zhuang H Xie L Feng X Liu J. Construction of a novel gene-based model for prognosis prediction of clear cell renal cell carcinoma. Cancer Cell Int (2020) 20:27. doi: 10.1186/s12935-020-1113-6 32002016 PMC6986036

[56] Augustin HG Koh GY Thurston G Alitalo K. Control of vascular morphogenesis and homeostasis through the angiopoietin-Tie system. Nat Rev Mol Cell Biol (2009) 10:165–77. doi: 10.1038/nrm2639 19234476

[57] Liao Z Yao H Wei J Feng Z Chen W Luo J. Development and validation of the prognostic value of the immune-related genes in clear cell renal cell carcinoma. Transl Androl Urol. (2021) 10(4):1607–19. doi: 10.21037/tau-20-1348 PMC8100830 33968649

[58] Shen CQ Liu J Wang JR Zhong XL Dong DH Yang XK. Development and validation of a prognostic immune-associated gene signature in clear cell renal cell carcinoma. Int Immunopharmacol. (2020) 81:106274. doi: 10.1016/j.intimp.2020.106274 32044664

[59] Ha M Son YR Kim J Park SM Hong CM Choi D. TEK is a novel prognostic marker for clear cell renal cell carcinoma. Eur Rev Med Pharmacol Sci (2019) 23:1451–8. doi: 10.26355/eurrev_201902_17102 30840266

[60] Nakayama T Saito K Kumagai J Nakajima Y Kijima T Yoshida S. Higher serum c-reactive protein level represents the immunosuppressive tumor microenvironment in patients with clear cell renal cell carcinoma. Clin Genitourin cancer. (2018) 16(6):e1151–e8. doi: 10.1016/j.clgc.2018.07.027 30213543

[61] Li JF Chu YW Wang GM Zhu TY Rong RM Hou J. The prognostic value of peritumoral regulatory T cells and its correlation with intratumoral cyclooxygenase-2 expression in clear cell renal cell carcinoma. BJU Int (2009) 103(3):399–405. doi: 10.1111/j.1464-410X.2008.08151.x 19021626

[62] Bruni D Angell HK Galon J. The immune contexture and immunoscore in cancer prognosis and therapeutic efficacy. Nat Rev Cancer. (2020) 20(11):662–80. doi: 10.1038/s41568-020-0285-7 32753728

[63] Pan Q Wang L Chai S Zhang H Li B. The immune infiltration in clear cell renal cell carcinoma and their clinical implications: A study based on TCGA and GEO databases. J Cancer. (2020) 11(11):3207–15. doi: 10.7150/jca.37285 PMC7097965 32231726

[64] Ma S Ge Y Xiong Z Wang Y Li L Chao Z. A novel gene signature related to oxidative stress predicts the prognosis in clear cell renal cell carcinoma. PeerJ (2023) 11:e14784. doi: 10.7717/peerj.14784 36785707 PMC9921988

[65] Zhang Y Zhou G Shi W Shi W Hu M Kong D. A novel oxidative-stress related lncRNA signature predicts the prognosis of clear cell renal cell carcinoma. Sci Rep (2023) 13(1):5740. doi: 10.1038/s41598-023-32891-z 37029263 PMC10082204

[66] Wu Y Zhang X Wei X Feng H Hu B Deng Z. A mitochondrial dysfunction and oxidative stress pathway-based prognostic signature for clear cell renal cell carcinoma. Oxid Med Cell Longev (2021) 2021:9939331. doi: 10.1155/2021/9939331 34868460 PMC8635875

