# Supplementary figures and images for: A novel oxidative stress-related genes signature associated with clinical prognosis and immunotherapy responses in clear cell renal cell carcinoma
Source: Front Oncol. 2023 Aug 3;13:1184841. doi: 10.3389/fonc.2023.1184841 (PMC10435754; doi:10.3389/fonc.2023.1184841)

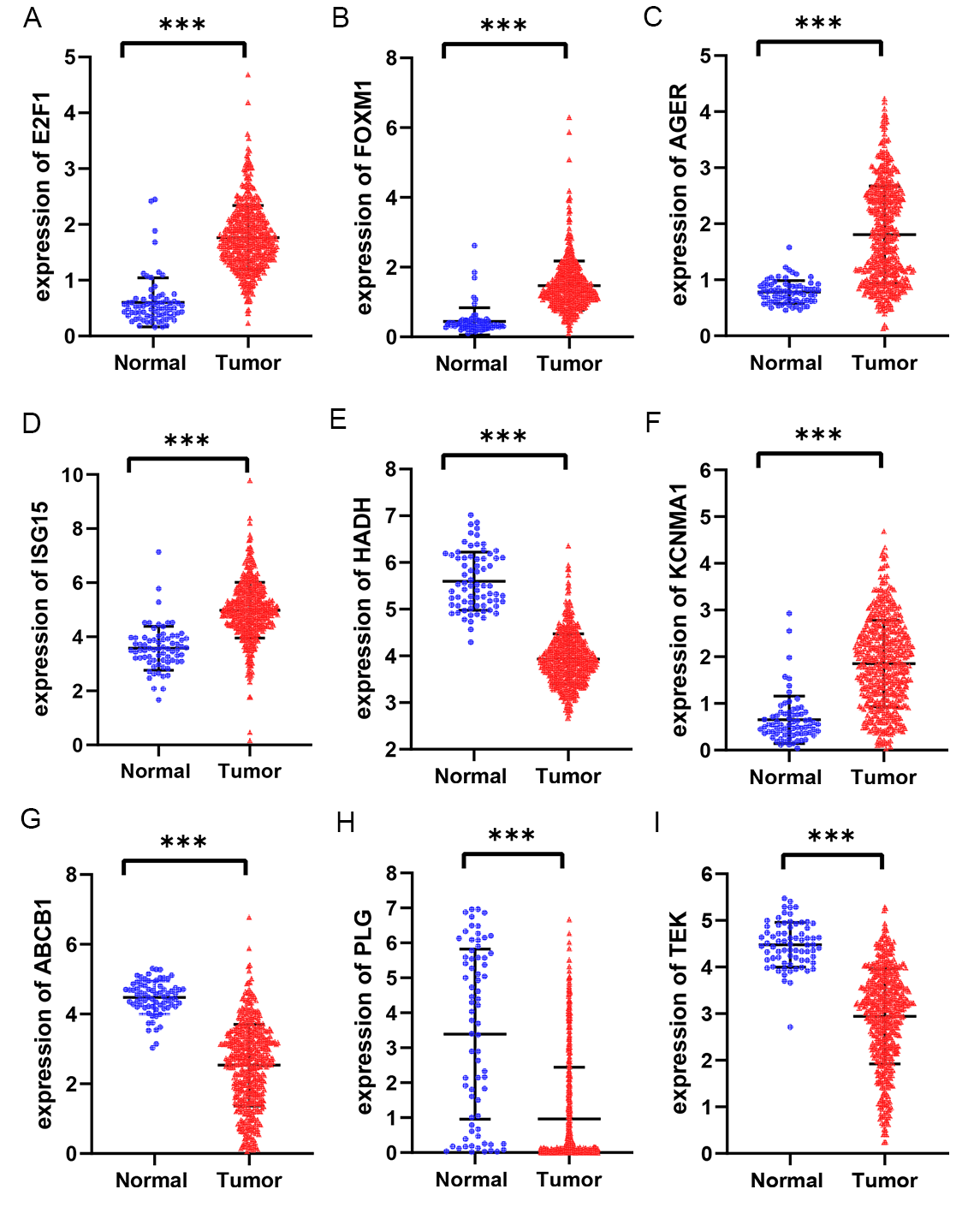

Supplement: Supplementary Figure 1 — (A–I) The expression levels of the signature genes (ABCB1, AGER, E2F1, FOXM1, HADH, ISG15, KCNMA1, PLG, and TEK) in ccRCC tissues compared with normal tissues in the TCGA data. [file Image_1.tif]

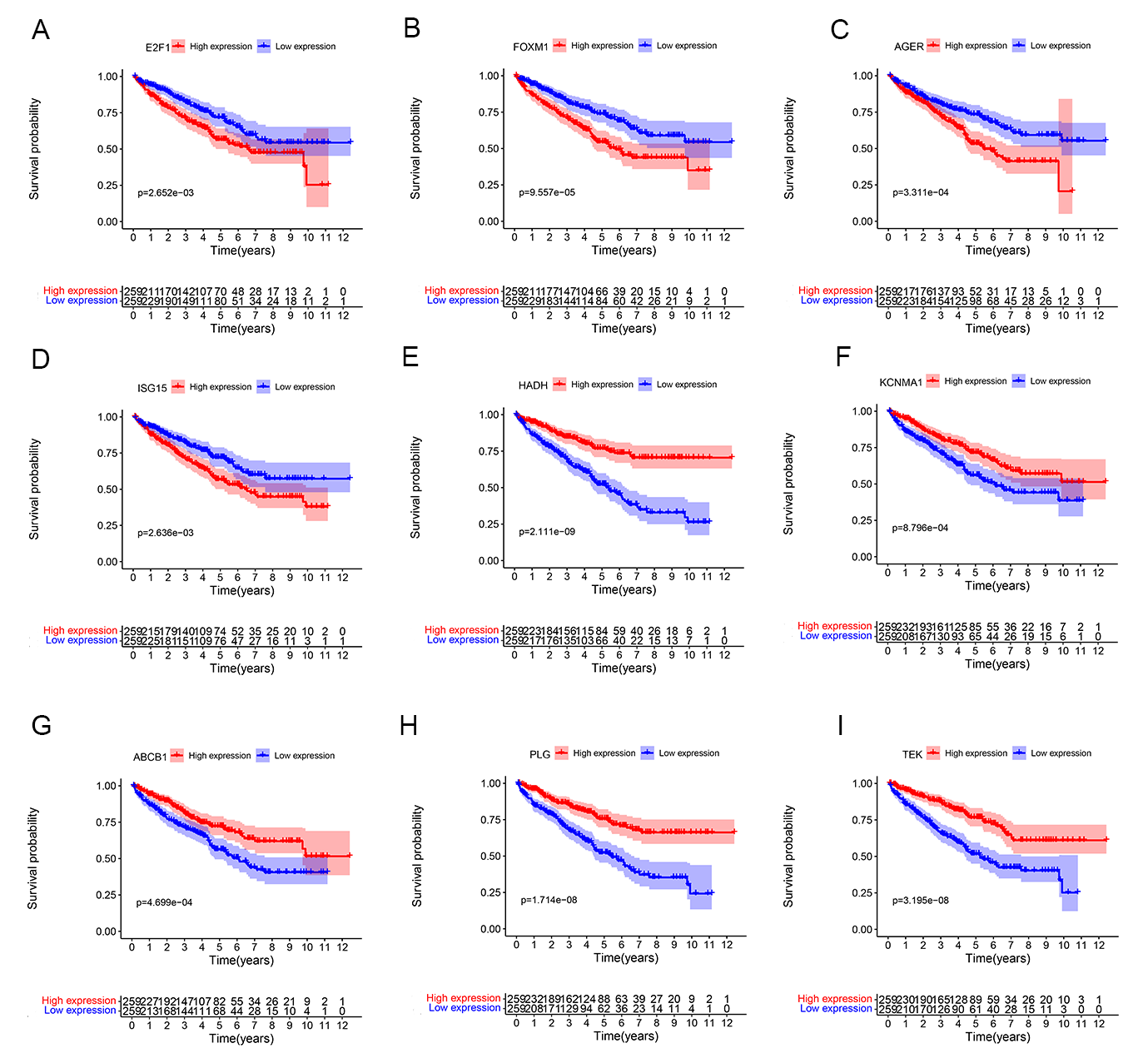

Supplement: Supplementary Figure 2 — (A–I) The survival plot for the signature genes (ABCB1, AGER, E2F1, FOXM1, HADH, ISG15, KCNMA1, PLG, and TEK) in the TCGA data. [file Image_2.tif]
